# RETRACTION: Effect of Platelet‐Rich Plasma vs. Standard Management for the Treatment of Diabetic Foot Ulcer Wounds: A Meta‐Analysis

**DOI:** 10.1111/iwj.70374

**Published:** 2025-03-21

## Abstract

**RETRACTION:** GongF. , ZhangY. , GaoJ. , LiX. , ZhangH. , MaG. , HuangY. , ZhangB. , and ZhaoF. , “Effect of Platelet‐Rich Plasma vs Standard Management for the Treatment of Diabetic Foot Ulcer Wounds: A Meta‐Analysis,” International Wound Journal 20, no. 1 (2023): 155–163, 10.1111/iwj.13858.35751432 PMC9797932

The above article, published online on 25 June 2022, in Wiley Online Library (http://onlinelibrary.wiley.com/), has been retracted by agreement between the journal Editor in Chief, Professor Keith Harding; and John Wiley & Sons Ltd. Following an investigation by the publisher, all parties have concluded that this article was accepted solely on the basis of a compromised peer review process. The editors have therefore decided to retract the article. The authors did not respond to our notice regarding the retraction.

**RETRACTION:** F. Gong , Y. Zhang , J. Gao , X. Li , H. Zhang , G. Ma , Y. Huang , B. Zhang , and F. Zhao , “Effect of Platelet‐Rich Plasma vs Standard Management for the Treatment of Diabetic Foot Ulcer Wounds: A Meta‐Analysis,” International Wound Journal 20, no. 1 (2023): 155–163, 10.1111/iwj.13858.35751432 PMC9797932

The above article, published online on 25 June 2022, in Wiley Online Library (http://onlinelibrary.wiley.com/), has been retracted by agreement between the journal Editor in Chief, Professor Keith Harding; and John Wiley & Sons Ltd. Following an investigation by the publisher, all parties have concluded that this article was accepted solely on the basis of a compromised peer review process. The editors have therefore decided to retract the article. The authors did not respond to our notice regarding the retraction.

